# Correction: DMT alters cortical travelling waves

**DOI:** 10.7554/eLife.64623

**Published:** 2020-11-09

**Authors:** Andrea Alamia, Christopher Timmermann, David J Nutt, Rufin VanRullen, Robin L Carhart-Harris

Alamia A, Timmermann C, Nutt DJ, VanRullen R, Carhart-Harris RL. 2020. DMT alters cortical travelling waves. *eLife* **9**:e59784. doi: 10.7554/eLife.59784.

Published 17, November 2020

David J Nutt was originally omitted from the author list, however he contributed to the conceptualization and supervision of the study as well as acquired funding and we believe he should be included as an author. All authors have agreed on the change.

**New authors list**: Andrea Alamia, Christopher Timmermann, **David J Nutt**, Rufin VanRullen, Robin L Carhart-Harris

**Original authors list**: Andrea Alamia, Christopher Timmermann, Rufin VanRullen, Robin L Carhart-Harris

Details for the omitted author:

**David J Nutt**

Centre for Psychedelic Research, Division of Psychiatry, Department of Brain Sciences, Imperial College London, London, United Kingdom

**Contribution:** Conceptualization, Data curation, Supervision, Funding acquisition

**Competing interests:** No competing interests declared

Revised contribution for Christopher Timmermann:

**Contributed equally with**: Andrea Alamia

Revised contribution for Andrea Alamia:

**Contributed equally with**: Christopher Timmermann

Revised funding declaration of Rufin VanRullen:

**CRCNS ANR-NSF (ANR-19-NEUC-0004)**

**ANITI (Artificial and Natural Intelligence Toulouse Institute, ANR-19-PI3A-0004) Research Chair**

Original funding declaration of Rufin VanRullen:

**CRCNS ANR-NSF (ANR-19-NEUC-0004)**

The article has been corrected accordingly.

